# Phylogeography of the South China Field Mouse (*Apodemus draco*) on the Southeastern Tibetan Plateau Reveals High Genetic Diversity and Glacial Refugia

**DOI:** 10.1371/journal.pone.0038184

**Published:** 2012-05-30

**Authors:** Zhenxin Fan, Shaoying Liu, Yang Liu, Lihuan Liao, Xiuyue Zhang, Bisong Yue

**Affiliations:** 1 Sichuan Key Laboratory of Conservation Biology on Endangered Wildlife, College of Life Sciences, Sichuan University, Chengdu, People’s Republic of China; 2 Sichuan Academy of Forestry, Chengdu, People’s Republic of China; 3 Key Laboratory of Bioresources and Ecoenvironment (Ministry of Education), Sichuan University, Chengdu, People’s Republic of China; Brigham Young University, United States of America

## Abstract

The southeastern margin of the Tibetan Plateau (SEMTP) is a particularly interesting region due to its topographic complexity and unique geologic history, but phylogeographic studies that focus on this region are rare. In this study, we investigated the phylogeography of the South China field mouse, *Apodemus draco*, in order to assess the role of geologic and climatic events on the Tibetan Plateau in shaping its genetic structure. We sequenced mitochondrial cytochrome *b* (cyt *b*) sequences in 103 individuals from 47 sampling sites. In addition, 23 cyt *b* sequences were collected from GenBank for analyses. Phylogenetic, demographic and landscape genetic methods were conducted. Seventy-six cyt *b* haplotypes were found and the genetic diversity was extremely high (π = 0.0368; *h* = 0.989). Five major evolutionary clades, based on geographic locations, were identified. Demographic analyses implied subclade 1A and subclade 1B experienced population expansions at about 0.052-0.013 Mya and 0.014-0.004 Mya, respectively. The divergence time analysis showed that the split between clade 1 and clade 2 occurred 0.26 Mya, which fell into the extensive glacial period (EGP, 0.5-0.17 Mya). The divergence times of other main clades (2.20-0.55 Mya) were congruent with the periods of the Qingzang Movement (3.6-1.7 Mya) and the Kun-Huang Movement (1.2-0.6 Mya), which were known as the most intense uplift events in the Tibetan Plateau. Our study supported the hypothesis that the SEMTP was a large late Pleistocene refugium, and further inferred that the Gongga Mountain Region and Hongya County were glacial refugia for *A. draco* in clade 1. We hypothesize that the evolutionary history of *A. draco* in the SEMTP primarily occurred in two stages. First, an initial divergence would have been shaped by uplift events of the Tibetan Plateau. Then, major glaciations in the Pleistocene added complexity to its demographic history and genetic structure.

## Introduction

The Tibetan Plateau is a recognized biodiversity hotspot [1]. Recently, a number of phylogeographic or phylogenetic studies have focused on the Tibetan Plateau due to its complex geologic history [2]–[8]. Historically, it uplifted several times, and the most intense uplift events were known as the Qingzang Movement (3.6-1.7 million years ago, Mya) and the Kun-Huang Movement (1.2-0.6 Mya) [9]–[11]. These events caused dramatic environmental changes: grasslands replaced forests and glaciers and deserts developed as the climate gradually became drier, colder and windier [12]. Thus, as a result of the uplift events, the plateau began to diversify topographically by the continual developed mountains and watercourse [13]. Moreover, it also caused its elevation to increase by up to 3000 m, which consequently increased the exposure of the endemic fauna to glacial events [5], [9]. So, Quaternary glacial cycles have been regarded as an important factor in promoting the diversification of species in this region [2], [14]. Although there are different opinions about the range and extent of glaciers on the plateau [15]–[17], ice-sheets during the most extensive glaciation (0.5-0.17 Mya) were undoubtedly several times larger than those found today [9]. Taken together, the current distribution of endemic organisms and their patterns of genetic differentiation should be greatly influenced by these geologic and climatic events.

Several studies have demonstrated that species distributed on the Tibetan Plateau retreated to the east side of the plateau during cold periods [2], [7], [18], [19]. Qu *et al.* [19] reported that both the twite (*Carduelis flavirostris*) and the black redstart (*Phoenicurus ochruros*) inhabited the northeastern margin of the Tibetan Plateau and maintained stable population sizes through glacial cycles. On the contrary, three other avian species (*Montifringilla adamsi*, *Pyrgilauda blanfordi* and *Eremophila alpestris*), mostly distributed on the inner region of the plateau, experienced population expansions after glacial retreat [19]. These results indicate that the east side of the plateau probably constituted a large refugium during the major glaciations in the Pleistocene.

**Figure 1:**
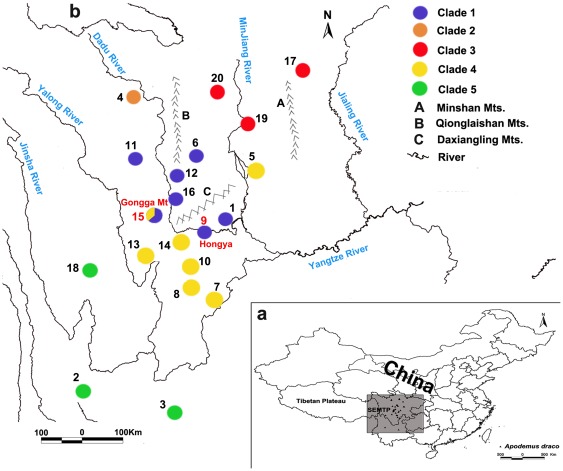
Sampling sites for the South China field mouse (*Apodemus draco*) in this study. (a) Map of China shows the study area of this study. The area in the shadow represents the range of the southeastern margin of the Tibetan Plateau, which is our study area. (b) Sampling sites for the South China field mouse (*Apodemus draco*) on the Tibetan Plateau. Populations are numbered as in Table 1. Major rivers and mountain are also shown. The distribution of observed major evolutionary clades in phylogenetic analyses is shown in different colors. The two glacial refugia (Gongga Mountain and Hongya County) were labeled in red color.

In this study, we focused on the southeastern margin of the Tibetan Plateau (SEMTP), including western Sichuan Province, northwestern Yunnan Province and eastern Xizang Autonomous Region (Tibet). The large rivers and mountains (e.g. Daxueshan Mountains, Shaluli Mountains, Gongga Mountains, Dadu River, Yalong River, and Jinsha River) in this region could be natural barriers for the postglacial colonization of small mammals (Fig. 1). The magnificent Gongga Mountain, with the highest peak of 7,556 m above the sea level, was an important glaciation centre and contemporary glaciers are still well developed [20]. Conversely, some previous studies indicated that Gonga Mountain region (GMR) was a suitable refuge during glacial cycles [21], [22]. Many ancient and endemic species inhabited in this region [21], [23]. But so far, none of the studies could provide detailed information.

**Table 1:** Sampling information of *Apodemus draco*.

Populations	Map label	Sample size	Sample names	Haplotypes	Latitude (^o^N)/Longitude (^o^E)	Altitude
Emeishan, Sichuan	1 (EMS)	1	EMS_1	EMS1	–	–
Dali, Yunnan	2 (DL)	1	DL_1	DL1	–	–
Kunming, Yunnan	3 (KM)	1	KM_1	KM1	–	–
Maerkang, Sichuan	4 (MEK)	13	Mar_1	MEK1	–	–
			Mar_2	MEK1	–	–
			Mar_3	MEK1	–	–
			Mar_4	MEK1	–	–
			Mar_5	MEK1	–	–
			Mar_6	MEK1	–	–
			Mar_7	MEK1	–	–
			Mar_8	MEK2	–	–
			Mar_9	MEK1	–	–
			Mar_10	MEK3	–	–
			Mar_11	MEK3	–	–
			Mar_12	MEK3	–	–
			Mar_13	MEK1	–	–
Zhongguo, Sichuan	5 (ZG)	7	Zhong_1	ZG1	–	–
			Zhong_2	ZG2	–	–
			Zhong_3	ZG3	–	–
			Zhong_4	ZG1	–	–
			Zhong_5	ZG4	–	–
			Zhong_6	ZG1	–	–
			Zhong_7	YX2	–	–
Jiajin Mountain, Sichuan	6 (JM)	16	Jiajin_1	JM4	30.84/102.752	2720
			Jiajin_2	JM5	30.843/102.689	1800
			Jiajin_3	JM6	30.843/102.689	3440
			Jiajin_4	JM7	30.84/102.752	2720
			Jiajin_5	JM8	30.886/102.655	3700
			Jiajin_6	JM4	30.909/102.648	1800
			Jiajin_7	JM9	30.84/102.752	2720
			Jiajin_8	JM10	30.84/102.752	2720
			Jiajin_9	JM11	30.909/102.648	1800
			Jiajin_10	JM12	30.886/102.655	3700
			Jiajin_11	JM2	30.601/102.753	3210
			Jiajin_12	JM2	30.601/102.753	2780
			Jiajin_13	JM3	30.614/102.798	2780
			Jiajin_14	JM2	30.629/102.855	3700
			Jiajin_15	JM2	30.629/102.855	3700
			Jiajin_16	JM1	30.629/102.855	1800
Jinyang, Sichuan	7 (JY)	3	Jinyang_1	JY2	27.808/103.187	3090
			Jinyang_2	ZG3	27.808/103.187	3090
			Jinyang_3	JY1	27.808/103.187	3090
Zhaojue, Sichuan	8 (ZJ)	1	Zhaojue_1	ZJ1	27.985/102.786	2140
Hongya, Sichuan	9 (HY)	9	Hongya_1	HY4	29.373/103.034	1880
			Hongya_2	HY1	29.373/103.034	1880
			Hongya_3	HY1	29.374/103.039	1820
			Hongya_4	HY2	29.373/103.034	1880
			Hongya_5	HY3	29.373/103.034	1880
			Hongya_6	HY1	29.373/103.034	1880
			Hongya_7	HY6	29.374/103.039	1820
			Hongya_8	HY5	29.374/103.039	1820
			Hongya_9	HY1	29.374/103.039	1820
Yuexi, Sichuan	10 (YX)	5	Yuexi_1	YX1	28.489/102.594	2130
			Yuexi_2	YX2	28.489/102.594	2130
			Yuexi_3	YX4	28.409/102.745	2720
			Yuexi_4	GM1	28.409/102.745	2720
			Yuexi_5	YX3	28.489/102.594	2130
Danba, Sichuan	11 (DB)	1	Danba_1	DB1	30.614/101.736	3610
Kangding, Sichuan	12 (KD)	1	Kangding_1	KD1	30.499/102.297	2400
Jiulong, Sichuan	13 (JL)	3	Jiulong_1	ZG3	29.222/102.958	2570
			Jiulong_2	JL1	28.675/101.610	2238
			Jiulong_3	JL2	28.675/101.610	2238
Shimian, Sichuan	14 (SM)	1	Shimian_1	SM1	29.025/102.391	2080
Gongga Mountain, Sichuan	15 (GM)	25	Gongga_1	GM1	29.351/101.873	2912
			Gongga_2	GM3	29.410/101.928	2602
			Gongga_3	GM2	29.396/101.971	2388
			Gongga_4	GM4	29.410/101.928	2602
			Gongga_5	GM9	29.702/102.017	2360
			Gongga_6	GM11	29.702/102.017	2360
			Gongga_7	GM7	29.574/101.999	3010
			Gongga_8	GM9	29.574/101.999	3010
			Gongga_9	GM9	29.574/101.999	3010
			Gongga_10	GM5	29.574/101.999	3010
			Gongga_11	GM9	29.576/102.002	3000
			Gongga_12	GM5	29.576/102.002	3000
			Gongga_13	GM9	29.698/102.002	3150
			Gongga_14	GM6	29.576/102.002	3000
			Gongga_15	GM8	29.574/101.999	3010
			Gongga_16	GM5	29.574/101.999	3010
			Gongga_17	GM9	29.574/101.999	3010
			Gongga_18	JM3	29.698/102.002	3150
			Gongga_19	GM10	29.698/102.002	3150
			Gongga_20	JM3	29.685/101.929	3460
			Gongga_21	JM3	29.574/101.999	3010
			Gongga_22	GM9	29.698/102.002	3150
			Gongga_23	JM3	29.698/102.002	3150
			Gongga_24	GM9	29.685/101.929	3460
			Gongga_25	GM5	29.574/101.999	3010
Erlang Mountain, Sichuan	16 (EM)	13	Erlang_1	EM2	29.852/102.263	2780
			Erlang_2	EM5	29.852/102.263	2780
			Erlang_3	EM5	29.852/102.263	2780
			Erlang_4	EM5	29.856/102.262	2780
			Erlang_5	EM6	29.856/102.261	2750
			Erlang_6	EM5	29.843/102.251	2450
			Erlang_7	HY1	29.865/102.278	3130
			Erlang_8	EM3	29.872/102.312	2250
			Erlang_9	EM4	29.873/102.309	2220
			Erlang_10	HY1	29.873/102.309	2220
			Erlang_11	EM5	29.879/102.345	1920
			Erlang_12	EM1	29.885/102.299	2450
			Erlang_13	HY1	29.885/102.299	2450
Qichuan, Sichuan	17 (QC)	10	Qingchuan_1	QC1	32.568/104.764	1450
			Qingchuan_2	QC4	32.581/104.755	1450
			Qingchuan_3	QC5	32.578/104.742	1500
			Qingchuan_4	QC2	32.568/104.764	1450
			Qingchuan_5	QC6	32.581/104.755	1450
			Qingchuan_6	QC7	32.581/104.755	1450
			Qingchuan_7	QC3	32.579/104.759	1450
			Qingchuan_8	QC8	32.579/104.759	1450
			Qingchuan_9	QC3	32.568/104.764	1450
			Qingchuan_10	QC9	32.918/105.478	1650
Daocheng, Sichuan	18 (DC)	4	Daocheng_1	DC1	28.451/100.345	3890
			Daocheng_2	DC2	28.451/100.345	3890
			Daocheng_3	DC3	28.451/100.345	3890
			Daocheng_4	DC4	28.452/100.347	3890
Maoxi, Sichuan	19 (MX)	5	Maoxian_1	MX1	31.694/103.905	1940
			Maoxian_2	MX2	31.694/103.905	1940
			Maoxian_3	MX1	31.694/103.905	1940
			Maoxian_4	MX1	31.694/103.905	1940
			Maoxian_5	MX1	31.694/103.905	1940
Heishui, Sichuan	20 (HS)	6	Heishui_1	HS4	32.244/103.153	2240
			Heishui_2	HS1	32.232/103.154	2470
			Heishui_3	HS2	32.323/103.129	2390
			Heishui_4	HS3	32.323/103.129	2390
			Heishui_5	HS3	32.241/103.153	2240
			Heishui_6	HS1	32.379/103.155	2540

We did not have the coordinates of the individuals in the first five populations because their sequences are from GenBank. Their references and GenBank accession numbers can be found in text.

In order to further assess the role of geologic and climatic events on the Tibetan Plateau in shaping the genetic structures of local fauna, we present a phylogeographic investigation of the South China field mouse (*Apodemus draco*). Small rodents are good candidates for inferring biotic history from contemporary patterns of genetic variation because their short generation time and relatively limited mobility [24]. This mouse is an endemic species to China, and is widely distributed from west to east, including in the Sichuan, Yunnan, Gansu, Qinghai, Shannxi, Hubei, Hunan, Fujian and Taiwan Provinces [23], [25]. However, the eastern part of the Tibetan Plateau was the centre of radiation of the genus *Apodemus* [18], [26]. One recent work sequenced the mitochondrial cytochrome *b* (cyt *b*) gene in six *A. draco* and 197 *A. ilex* with samples from Yunnan-Guizhou Plateau in China, they reported that *A. ilex* should be a valid species rather than synonym of *A. draco* [27]. They also indicated that the phylogenetic pattern of *A. ilex* was closely related to the complex geographical structures in southwest China [27].

The cyt *b* gene sequence has been extensively and successfully used in phylogeographic studies for rodents [27]–[33]. Therefore, the nucleotide sequences of the cyt *b* gene were applied to investigate the genetic variation and evolutionary history of *A. draco*. We examined: (1) the relationship between the genetic structure of *A. draco* and geologic or climatic events on the SEMTP, and (2) whether the SEMTP, especially the GMR, potentially functioned as a refuge during the Quaternary period. Details of the historical demography of *A. draco* revealed in this study could provide new insights into how this rodent and other small mammals in this region responded to the geologic or climatic events on the SEMTP.

## Materials and Methods

### Ethics Statement

We only used wild rodents in this study. All samples were obtained following the regulations for the implementation of China on the protection of terrestrial wild animals (State Council Decree [1992] No. 13). All our observational and field studies and lab work were approved by Wildlife Protection Office, Sichuan Provincial Forestry Departments (China) and by the Ethics Committee of Sichuan University, China.

### Samples and DNA Extraction

One Hundred and three South China field mice were collected from 47 sampling sites in Sichuan Province (Table 1). In addition, 23 cyt *b* sequences from GenBank were retrieved (GenBank Accession Nos. AB096825, AB109397, AM945800-AM945819, AY389007), which were collected from an additional six sampling sites in the Sichuan and Yunnan Provinces (Table 1) [18], [34], [35]. We combined the very near sampling sites (which located at the same mountain or village) into one population. In total, we used 126 individuals from 20 populations (Table 1). Cyt *b* sequences from more individuals were in GenBank, but the length of the sequences was significantly shorter (319 bp or 402 bp) than our target sequences (1050 bp), thus we did not include them here. Detailed information on sampling localities is shown in Fig. 1 and Table 1. Voucher specimens were deposited in the Museum of the Sichuan Academy of Forestry. Total genomic DNA was extracted from muscle or liver tissues preserved in 95% ethanol using standard procedures [36].

### Amplification and Sequencing

Polymerase chain reaction (PCR) was used to amplify the sequences of cyt *b* with the universal primers L14724/H15915 [37]. PCR conditions included an initial denaturation for 5 min at 95°C, 35 cycles of denaturation for 40 s at 94°C, 45 s annealing at 50–60°C, and a 1 min extension at 72°C followed by a final extension at 72°C for 10 min. The amplification was performed in 25 µl reaction volumes with 2.5 µl of 10×^EX^ *Taq* buffer (Mg^2+^ Free; TaKaRa Biotech), 1.5 µl dNTP (2.5 mM each), 2.0 µl MgCl_2_ (25 mM), 1 µl of each primer (10 uM), 0.2 µl ^EX^ *Taq* polymerase (5 U/µl, TaKaRa Biotech), and approximately 200 ng total genomic DNA as template. PCR products were sequenced from both directions with the same PCR primers in an ABI PRISM 3730 DNA sequencer (Applied Biosystems, Inc.).

### Genetic Diversity and Protein Sequences

We aligned the sequences in software MEGA 4.0 [38]. Nucleotide saturation was tested by plotting transitions and transversions against the JC69 distance using DAMBE [39]. Haplotype diversity (*h*) and nucleotide diversity (π) were estimated using DnaSP Ver. 5 [40]. Genetic differentiation between populations was evaluated by estimating pairwise values of *F_ST_* with the software Arlequin 3.1 [41]. The significance was assessed by 1000 permutations. Populations contain only one sample were excluded in this analysis. Pairwise comparisons were used as genetic distances in the following analyses, such as isolation by distance. McDonald–Kreitman test [42] was used to test the overall selection on cyt *b* on the website (http://mkt.uab.es) [43]. In this analysis, the numbers of synonymous and nonsynonymous substitutions in cyt *b* of *A. draco* and *A. latronum* (Nos. GU908339-GU908394) were estimated and their ratio was compared for each species with the same ratio for fixed mutations between the species. The cyt *b* sequences of *A. latronum* were used here due to *A. latronum* was the sister taxon to *A. draco*.

### Phylogenetic Analysis

Two different methods of phylogenetic analysis were used to reconstruct phylogenetic relationships among cyt *b* haplotypes: Bayesian inference (BI) and maximum parsimony (MP). *Apodemus latronum* (GU908426-GU908428, GU908433, GU908436) was used as outgroup due to it was the sister taxon to *A. draco*. In order to avoid the possible bias, *A. flavicollis* (AB032853) and *A. sylvaticus* (AB033695) were also chosen as outgroups. The best fit model of DNA substitution was obtained using MrModeltest2.2 [44] under the Akaike Information Criterion (AIC). BI was performed with MrBayes 3.1.2 [45]. For BI, we used two different datasets, partitioned and non-partitioned. For partitioned dataset, the nucleotides sequences were partitioned by codon position. In total, three partitions were given; one each for codon positions 1, 2, and 3 cyt *b*. Posterior distributions were obtained by the Markov Chain Monte Carlo (MCMC) method with one cold chain and three heated chains for 10,000,000 generations and sampled every 1000 generations. The first 25% were discarded as a conservative burn-in and the remaining samples were used to generate a 50% majority rule consensus tree. PAUP* 4.0 b 10 [46] was used to perform MP analysis. A heuristic search strategy was employed with the tree bisection and reconnection (TBR) branch swapping algorithm, random addition of taxa and 1000 replicates per search. Supports for branches in the MP trees were tested by bootstrap analysis with 1000 replicates.

Since the haplotype network could better detect the relationship among haplotypes, we also constructed a median-joining network (MJN) approach to depict relationships among the cyt *b* haplotypes of *A. draco* using Network 4.6.0.0 [47].

### Landscape Analyses

We used analysis of molecular variance (AMOVA) to estimate the geographical pattern of population subdivision using different grouping options [48]. To test for the correlation between genetic differentiation and geographic distance, we performed a Mantel test [49] and a spatial autocorrelation analysis. In Mantel test, we used the Euclidean distance between sampling localities, based on GPS coordinates, as the geographic distance. Because we did not have the coordinates of the samples from GenBank, we approximated distance using mountains or county locality information listed in the corresponding papers [18], [34], [35]. We used three different datasets to perform the Mantel test. First one included 13 populations (exclude populations that only contain one sample); second one further excluded all the samples from other studies since we did not have their exact coordinates. Third one only contained the populations in clade 1 (Jiajin, Gongga and Erlang Mountains, and Hongaya county). The significance of the Mantel test was determined by 1,000 permutations using the isolation by distance (IBD) web service v3.16 (http://ibdws.sdsu.edu) [50]. The spatial autocorrelation analysis was performed in Allele in Space (AIS) [51]. The spatial autocorrelation algorithm calculates the statistic *Ay*, the average genetic distance between individuals in distance class *y*. Thus, Ay can be interpreted as the average genetic distance between pairs of individuals that fall within distance classes [51]. In order to ensure that the arbitrary defined distance class size had no effect on result, analyses were performed using 5, 10 and 15 distance classes, respectively. We used a randomisation procedure consisting of 5,000 replicates to identify distance classes where average genetic distances were significantly larger or smaller than random expectations. Because this algorithm uses genetic and spatial data for every individual, we preformed this analysis on different data set, all individuals and individuals without GenBank samples based on the same reason we explained above.

### Molecular Dating

Divergence times among the observed mitochondrial clades on phylogenetic tree were estimated by BEAST v1.7.1 [52] using a Bayesian coalescence approach. We also used partitioned (by codon positions) and non-partitioned data. For partitioned data, we had two different partitioned datasets here. The first one had two partitions (pos1+pos2, pos3), and the second one had three (pos1, pos2, pos3). We used an uncorrelated lognormal relaxed clock with the GTR+I+G model of substitution. All analyses were performed using a coalescent-constant size process of diversification. The prior for mean mutation rate was specified as a lognormal distribution, with a mean of 0.02, log (standard deviation) of 0.56 and offset of 0.0172. This distribution covered the range from 2% to 8% substitutions per site per Myr (million years), with the 95% highest posterior density (HPD) range of 2.4% to 6.01% per Myr. We used this prior because the standard divergence rate for the mammalian cyt *b* gene is estimated at 2% per Myr [53], and the evolutionary rate of cyt *b* in genus *Apodemus* is estimated at 2.4% per Myr [34], [54]. However, a number of studies [28], [29], [55], [56] indicate that the cyt *b* gene of rodents in general evolved several times faster than the standard rate. Moreover, Fan *et al.* [22] applied a rate of 2.4–8% per Myr for a phylogeographic analysis of another field mouse, *A. latronum* in their study. Monophyly of clades was not enforced, and runs were initiated on random starting trees. The MCMC chain was run for 50 million generations, sampling every 1,000 generations. The convergence of the chains to stationary was checked using Tracer 1.5 [57] and the first 25% were discarded as burn-in. The final molecular clock tree was produced in TreeAnnotator 1.7.1 [58] using the mean as node heights. Finally, Figtree v1.3.1 [59] was used to visualize the results. Although the divergence times were only rough estimates here, it will provide useful information when we discuss *A. draco*’ s divergence.

### Historical Demography

The demographic history was tested by mismatch distributions and neutrality tests based on the cyt *b* sequences. First, two tests of selective neutrality were calculated for each set (whole sample and each observed phylogenetic clade). Fu’s *Fs* [60] was calculated in Arlequin 3.1 [41], and Ramos-Onsins and Rozas’s R_2_ statistics [61] was calculated in DnaSP Ver. 5 [40]. Fu’s *Fs* and R_2_ were applied due to the fact that they have been suggested as the most powerful tests for detecting sudden population growth or contractions [61], [62]. Fu’s *Fs* is more powerful for large population sizes, whereas R_2_ works well for small ones [61]. The sum of squared deviations (SSD) and the raggedness index (*r*) [63] were also estimated in Arlequin 3.1 to determine whether the sequences deviated significantly from a model of population expansion. Significance of all the statistics was calculated using 1,000 coalescent simulations with the software Arlequin 3.1 and DnaSP Ver. 5. Mismatch analyses [64] were carried out using DnaSP Ver. 5. The time of possible population expansions (t) was calculated according to τ = 2 ut [65], where τ was the model of the mismatch distribution and u was the mutation rate of the DNA sequence. The value u was derived from u = µk, where μ was the mutation rate per nucleotide and k was the number of nucleotides. We used an estimated evolutionary rate of 2–8% per Myr for cyt *b* (rationale is provided in previous section).

## Results

### Sequence Information

We generated 103 cyt *b* gene sequences each consisting of 1050 sites (GenBank Accession Nos. HM162766 - HM162833, JQ424902 - JQ424910). With sequences from the additional 23 individuals, we recovered 76 haplotypes from 126 individuals, and the overall *h* was 0.982, while the π was 0.0381 (Table 2). Saturation analysis did not show any evidence of saturation, so our sequences were used for further analyses. We were confident that the cyt *b* sequences obtained in our study represented the mitochondrial cytochrome *b* gene since all cyt *b* sequences could be translated into amino acid sequences without interruption and closely matched the previously published cyt *b* sequence for *A. draco.* The McDonald–Kreitman test did not provide overall evidence for positive selection on the cyt *b* gene [42], [43]. The value of P_n_/P_s_ (polymorphism) was larger than D_n_/D_s_ (divergence), and the difference between them was not significant (Table 3).

**Table 2:** Genetic diversity indices and demographical analyses for cyt *b* sequences in *Apodemus draco*.

mtDNA clades	*n*	*H*	*h* (SD)	π (SD)	Fu’s *Fs*	R_2_	Mismatch distribution	*r*	SSD
Clade 1	56	31	0.951	0.0066	**−12.969**	0.065	**–**	0.008	0.006
Subclade 1A	28	15	0.902	0.0025	**−8.201**	**0.064**	unimodal	0.020	0.001
Subclade 1B	18	7	0.752	0.0016	**−2.068**	**0.104**	unimodal	0.110	0.023
Subclade 1C	10	9	0.978	0.0042	**−4.067**	0.139	multimodal	0.069	0.016
Clade 2	13	3	0.500	0.0010	0.930	0.164	multimodal	0.679	0.173
Clade 3	21	15	0.957	0.0050	**−5.779**	**0.081**	multimodal	0.016	0.009
Clade 4	30	21	0.968	0.0084	**−6.044**	0.112	multimodal	0.013	0.008
Clade 5	6	6	1.000	0.0064	**−**1.606	0.257	multimodal	0.071	0.046
Total	126	76	0.982	0.0381	**−**8.413	0.108	–	0.002	0.012

Number of individuals (*n*), number of haplotypes (*H*), haplotype diversity (*h*), nucleotide diversity (π), Fu’s *Fs*, Ramos-Onsins and Rozas’s R_2_ statistics (R_2_), the shape of mismatch distribution, the sum of squared deviations (SSD) and raggedness indexes (*r*) are shown. Numbers in bold indicate statistically significant genetic differentiation (*P*<0.05).

**Table 3:** The result of McDonald-Kreitman test for cyt *b* gene of *Apodemus draco* (N = 126) and *A. latronum* (N = 68).

	Polymorphism	Divergence
Non-Neutral	66	6 (6.03)
Neutral	243	45 (50.94)
Ratio	0.27	0.13 (0.12)
Neutrality Index	2.037 (2.294)	
χ2 *P*-value	0.112 (0.06)	

Values corrected by Jukes and Cantor (1969) are given in parentheses.

### Phylogeographic Structure

**Figure 2:**
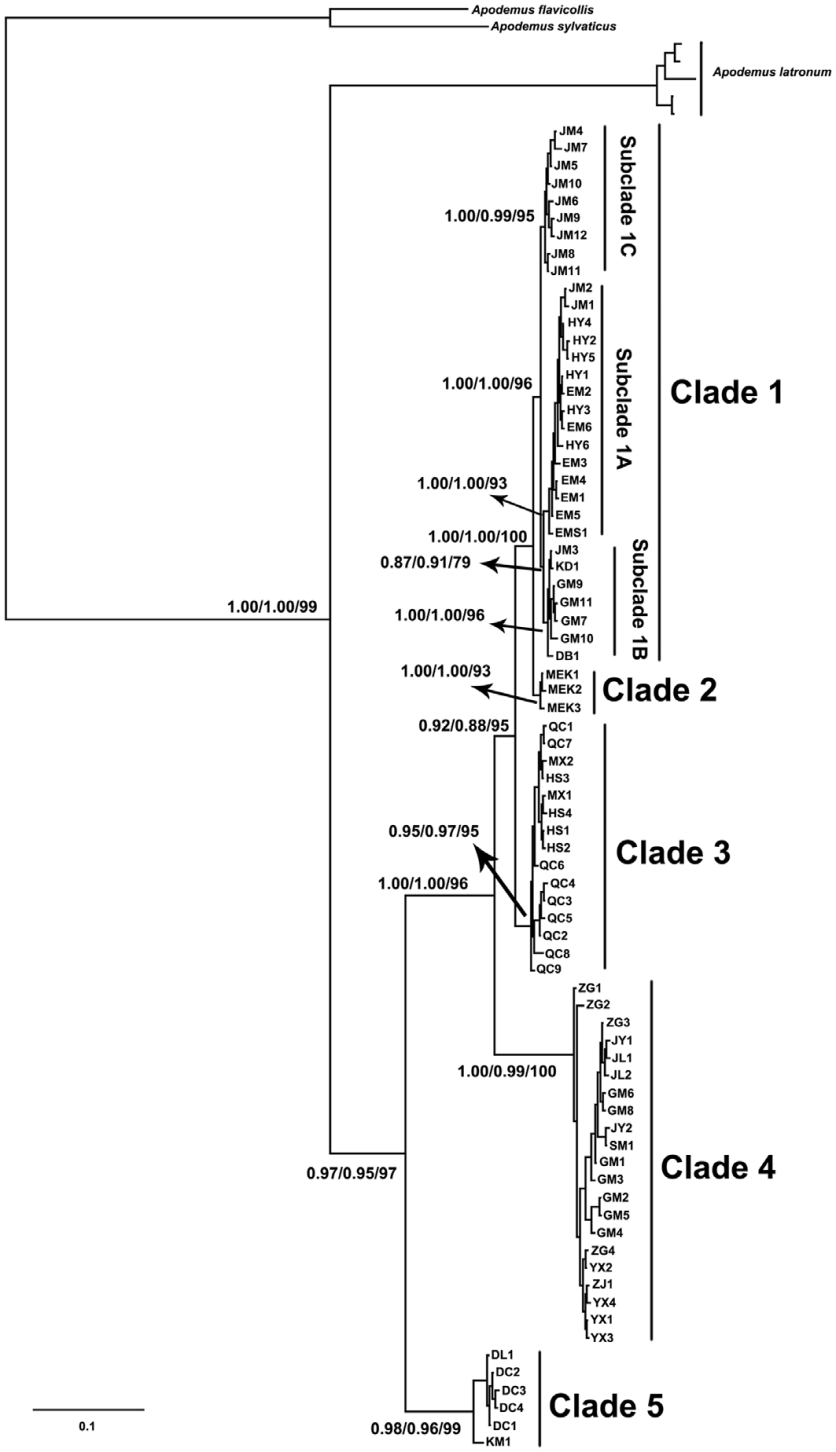
Fifty percent majority rule consensus tree from Bayesian analysis of *Apodemus draco* based on all the haplotypes of cyt *b* data. Numbers represent node supports inferred from Bayesian posterior probability of non-partitioned data and partitioned data, and maximum parsimony bootstrap analyses, respectively. Only values of the main evolutionary clades are shown. The haplotype names can be found in Table 1.

Based on the AIC test, the GTR+I+G model was chosen for the cyt *b*. For partitioned data, different models of sequence evolution were detected (pos1: SYM+I; pos2: HKY+I; pos3: GTR+I+G). The 50% majority consensus tree based on BI was shown in Fig. 2. MP tree recovered the same tree topology and was not shown. The phylogenetic trees based on partitioned and non-partitioned datasets exhibited the same topology.

Five major evolutionary clades, based on geographic locations, were identified: Maerkang (clade 2), northern Sichuan (including Qingchuan, Maoxian and Heishui counties, clade 3), southern Sichuan (including Jinyang, Zhaojue, Yuexi, Jiulong and Shimian counties and the Gongga Mountain and Zhongguo, clade 4), and Daocheng county and Yunnan Province (clade 5) (Fig. 2). Haplotypes from Jiajin Mountain, Emei Mountain, Erlang Mountain, Gongga Mountain, and Hongya, Danba and Kangding counties formed clade 1. In this clade, three subclades were identified. Haplotypes from Jiajin Mountain clustered together with samples from Erlang Mountain, Emei Mountain and Hongya county, and then they formed subclade 1A. One haplotype (JM3) from Jiajin Mountain fell into subclade 1B, which was clustered with individuals from Gongga Mountain, Danba and Kangding Counties. Then, the remaining haplotypes from Jiajin Mountain (11 individuals, nine haplotypes) formed subclade 1C. Thus, the haplotypes from Gongga Mountain distributed in both clade 1 and clade 4, and samples from Jiajin Mountain appeared in all the subclades of clade 1.

**Figure 3:**
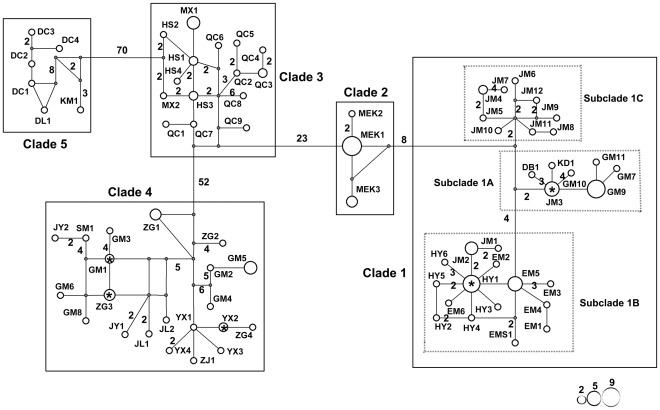
Median-joining network of all cyt *b* haplotypes found in *Apodemus draco*. The missing haplotypes in the network are represented by gray dots. Circle sizes are proportional to the number of individuals sharing the same haplotypes. Each mutation step is shown as a short line connecting neighboring haplotypes, and numbers of mutations between haplotypes are indicated near branches if it is greater than one. Haplotype designations can be found in Table 1. Evolutionary clades correspond to the five major clades and three subclades in Fig. 2. Haplotypes from different populations are marked as *.

The haplotype network also revealed five clades separated by 9 to 73 mutational steps (Fig. 3), which was consistent with the phylogenetic trees. The subclades within clade 1 only separated by four to six mutational steps, which exhibited very close relationships. Moreover, haplotype YX2 was found at samples both from Yuexi and Zhongguo, HY1 was identified in individuals from Hongya and Erlang Mountain, and JM3 was observed in individuals from Jiajin Mountain and Gongga Mountain.

### Genetic Variation and Landscape Genetic Analyses

The *F*_ST_ values for pairwise populations comparisons ranged from −0.026 to 0.985. Of the pairwise values obtained, only 3 of the 78 were nonsignificant (Table 4). The AMOVA analysis showed a very strong differentiation among different populations or evolutionary clades, and showed that among populations or clades variation accounted for 66% (*p*<0.001) or 86% (*p*<0.001) of the overall variation. However, when we focused on the populations in clade 1, the result exhibited that among populations variation only accounted for 26% of the overall variation, but 48% of the overall variation was contributed by variation among samples (Table 5).

**Table 4:** Table **4.** Pairwise *F*_ST_ values among the 13 populations surveyed of *Apodemus draco*.

	4 (MEK)	5 (ZG)	6 (JM)	7 (JY)	9 (HY)	10 (YX)	13 (JL)	15 (GM)	16 (EM)	17 (QC)	18 (DC)	19 (MX)
5 (ZG)	**0.959**	–										
6 (JM)	**0.700**	**0.907**	–									
7 (JY)	**0.970**	**0.406**	**0.907**	–								
9 (HY)	**0.904**	**0.950**	**0.379**	**0.958**	–							
10 (YX)	**0.969**	0.059	**0.911**	**0.415**	**0.960**	–						
13 (JL)	**0.981**	**0.470**	**0.918**	-0.026	**0.972**	**0.560**	–					
15 (GM)	**0.379**	**0.426**	**0.287**	**0.386**	**0.312**	**0.416**	**0.412**	–				
16 (EM)	**0.919**	**0.961**	**0.421**	**0.970**	**0.058**	**0.970**	**0.980**	**0.348**	–			
17 (QC)	**0.884**	**0.907**	**0.794**	**0.909**	**0.872**	**0.913**	**0.923**	**0.429**	**0.894**	–		
18 (DC)	**0.985**	**0.954**	**0.934**	**0.957**	**0.978**	**0.964**	**0.979**	**0.666**	**0.984**	**0.942**	–	
19 (MX)	**0.958**	**0.939**	**0.822**	**0.952**	**0.946**	**0.955**	**0.979**	**0.394**	**0.959**	**0.310**	**0.984**	–
20 (HS)	**0.956**	**0.941**	**0.828**	**0.954**	**0.945**	**0.956**	**0.978**	**0.409**	**0.958**	**0.325**	**0.983**	−0.004

Populations contain only one sample are exclude in this analyses.

Numbers in bold indicate statistically significant genetic differentiation (*P*<0.05). Populations are numbered as in Table 1.

**Table 5:** Table **5.** Results of hierarchical analyses of molecular variance (AMOVA) conducted using different grouping options.

Groups compared	No. groups	Source of variation	d.f.	Percentage variation (%)	*P* value	*F* statistics
All populations	13	Among populations	12	66.52	0.000	*F*_CT_ = 0.665
		Among sites within populations	21	13.87	0.016	*F*_SC_ = 0.414
		Among samples within sites	85	19.61	0.000	*F*_ST_ = 0.804
Populations in clade 1* ^#^	4	Among populations	3	26.35	0.019	*F*_CT_ = 0.264
		Among sites within populations	14	25.49	0.017	*F*_SC_ = 0.346
		Among samples within sites	45	48.15	0.000	*F*_ST_ = 0.518
Main clades*	5	Among clades	4	86.37	0.000	*F*_CT_ = 0.864
		Among populations within clades	9	7.06	0.000	*F*_SC_ = 0.518
		Among samples within populations	105	6.58	0.000	*F*_ST_ = 0.934
Subclades in clade 1*	3	Among subclades	2	70.73	0.016	*F*_CT_ = 0.707
		Among populations s within subclades	3	10.76	0.000	*F*_SC_ = 0.368
		Among samples within populations	47	18.51	0.000	*F*_ST_ = 0.815

* Populations contain only one sample are exclude in all the analyses Main evolutionary clades and subclades in clade 1 are observed in our phylogenetic trees.

# Jiajin Moutain, Erlang Moutain, Gongga Mountain, Hongya county.

**Figure 4:**
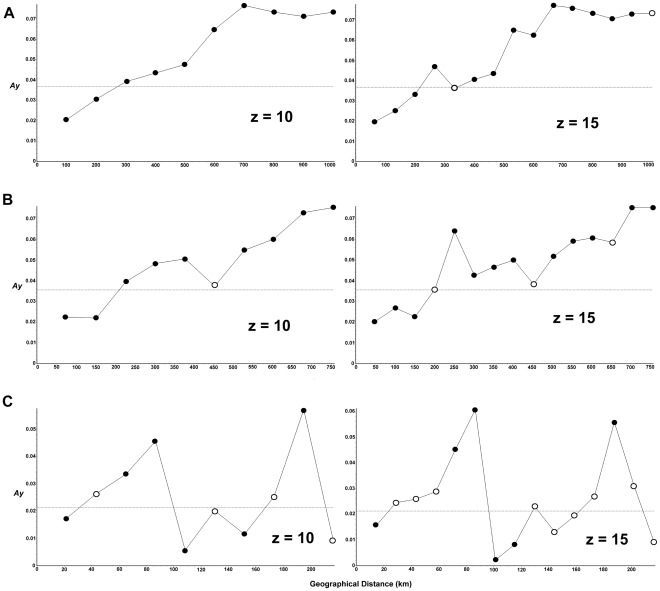
Results of spatial autocorrelation analyses of cyt *b* sequences in *Apodemus draco*. Analyses were performed using different distance classes (5, 10 and 15). Only the results of z = 10 and 15 distance classes were shown. Black circles represent values significantly different (*p*<0.05) from the mean *Ay*. (a) All individuals. (b) Exclude all the GenBank sequences due to we did not have their coordinates. (c) Individuals in clade 1.

Three different datasets were applied to perform the Mantel test. A significant correlation between genetic (*F*_ST_) and geographic distances was detected in first two datasets (13 populations: *p* = 0.0009, *r* = 0.4122; 11 populations: *p* = 0.0006, *r* = 0.4944), indicating high levels of geographical structure for *A.draco*. But for the dataset only including 4 populations in clade 1, the result was non-significant (*p* = 0.3800, *r* = 0.1831). Spatial autocorrelation analysis (SAA) also found a significant relationship between genetic and geographic distances in different distance classes (5, 10 and 15, only show the results of 10 and 15). In analyses performed using 10 distance classes, both the whole individuals dataset and without GenBank samples dataset were significant smaller than random expectations over the first two shortest distance classes (geographic distance up to ∼200 and 150 km, respectively). Then, when the geographic distance increased (start from 300 and 200 km, respectively), both the datasets yielded significant values of *Ay* that were higher than random expectations. Similar results were obtained when 5 and 15 distance classes were used for analyses (Fig. 4). Different results were observed when we performed the analyses with individuals only in clade 1. First, it yielded significant smaller value than random expectations at the shortest distance class (∼20 km). Then, significant higher values than random expectations were observed at around 60–80 km. But this dataset showed significant smaller and higher values again at around 100 km and 180 km, respectively (Fig. 4).

### Estimation of Divergence Times

Although we applied three different datasets to estimate the divergence time, we got the very similar results. Therefore, we described and discussed the divergence times based on the three partitions dataset (Table 6). The divergence time between clade 5 and the remaining clades occurred during the Pliocene/Pleistocene boundary at approximately 2.20 Mya, although the range of the 95% HPD interval spanned the period from the late Pliocene through the middle Pleistocene, between 3.80 and 0.87 Mya (Table 6). The divergence between clade 4 and its sister clade and clade 3 and its sister clade occurred in the middle Pleistocene at 1.22 Mya (95% HPD 0.50-2.06 Mya) and 0.55 Mya (95% HPD 0.22-0.96 Mya), respectively. The divergence between clade 1 and clade 2 was the youngest split, which also occurred in the middle Pleistocene at 0.26 Mya (95% HPD 0.09-0.45 Mya). The splits within clade 1 occurred during the late/middle Pleistocene boundary at 0.13 Mya and 0.16 Mya.

**Table 6:** Result of the Bayesian coalescent-based estimation of divergence time among different evolutionary clades/subclades and the time to the most recent common ancestor (*t*_MRCA_) of different evolutionary clades/subclades.

	Divergence time estimate (Mya)		
Evolutionary clades	Non-partition	Two partitions	Three partitions
Clade 1 vs. Clade 2	**0.2539** (0.0903–0.4742)	**0.2544** (0.096–0.4648)	**0.2566** (0.0942–0.4530)
Clade 3 vs. Clade 1 - Clade 2	**0.5515** (0.1965–0.9945)	**0.5490** (0.2089–0.9809)	**0.5463** (0.2215–0.9607)
Clade 4 vs. Clade 1 - Clade 2 - Clade 3	**1.1659** (0.4652–1.9848)	**1.1958** (0.4755–2.0324)	**1.2180** (0.4971–2.0552)
Clade 5 vs. Clade 1 - Clade 2 - Clade 3 - Clade 4	**2.0905** (0.8492–3.4836)	**2.1829** (0.8575–3.8453)	**2.1952** (0.8733–3.8037)
Subclade 1A vs. Subclade 1B	**0.1302** (0.0488–0.2382)	**0.1311** (0.0519–0.2341)	**0.1312** (0.0523–0.2314)
Subclade 1C vs. Subclade 1A - Subclade 1B	**0.1612** (0.0590–0.2956)	**0.1628** (0.0653–0.2929)	**0.1635** (0.0644–0.2862)
	*t*_MRCA_		
Clade 1	**0.1612** (0.0590–0.2956)	**0.1628** (0.0653–0.2929)	**0.1635** (0.0644–0.2862)
Clade 2	**0.0444** (0.0090–0.1086)	**0.0443** (0.0092–0.1085)	**0.0447** (0.0107–0.1071)
Clade 3	**0.1251** (0.0419–0.2474)	**0.1272** (0.0452–0.2450)	**0.1283** (0.0455–0.2404)
Clade 4	**0.2098** (0.0747–0.3887)	**0.2080** (0.0809–0.3881)	**0.2093** (0.0778–0.3720)
Clade 5	**0.2038** (0.0571–0.4302)	**0.2217** (0.0655–0.4540)	**0.2211** (0.0652–0.4558)
Subclade 1A	**0.0788** (0.0266–0.1490)	**0.0800** (0.0282–0.1495)	**0.0805** (0.0294–0.1497)
Subclade 1B	**0.0681** (0.0181–0.1278)	**0.0622** (0.0189–0.1231)	**0.0625** (0.0206–0.1231)
Subclade 1C	**0.0780** (0.0222–0.1541)	**0.0793** (0.0242–0.1528)	**0.0799** (0.0257–0.1554)

Results based on non-partition and partitioned by each codon were shown (two partitions: pos1+pos2, pos3; three partitions: pos1, pos2, pos3). Mean values are shown in bold and 95% HPD are shown in brackets.

### Historical Demography

We did not include the data sets of whole sample and clade 1 in mismatch distribution because the phylogenetic trees indicate they exhibit a high level of population structure. But the non-significant values of Fu’s *Fs* and Ramos-Onsins and Rozas’s R_2_ indicated that the whole population maintained a stable population size. The mismatch distribution analysis indicated a ragged and multimodal distribution (Table 2), suggesting a relatively constant population size for the clades 2, 3, 4 and 5, this result was also supported by non-significant values of Fu’s *Fs* and R_2_ tests for clades 2 and 5 (Table 2). However, Fu’s *Fs* for clade 4, and Fu’s *Fs* and R_2_ for clade 3 showed significant values. Thus, considering the multimodal distribution in mismatch distribution of clades 3 and 4, it was hard to conclude whether they experienced a population expansion.

The Subclade 1C showed multimodal distribution and non-significant value in R_2_, but a significant Fu’s *Fs* value. This inconsistent pattern maybe caused by single sampling region of this subclade. We did not calculate the possible expansion time for clade1, because historical expansion in subclade 1A and subclade 1B was also supported by unimodal mismatch distributions and significant negative values of all the neutrality tests (Table 2). The values of τ were different in subclades 1A and 1B, indicating that clade 1 did not simply experience a single expansion. Therefore, we calculated the possible expansion times for subclades 1A and 1B instead of clade 1. Here, τ = 2.166 for subclade 1A and τ = 0.598 for subclade 1B, generation time was one year, and k was 1050. Thus, subclade 1A and subclade 1B underwent expansions at 0.052-0.013 Mya and 0.014-0.004 Mya, respectively.

## Discussion

Sakka *et al.* [35] studied the phylogeography of four *Apodemus* species (*A.draco*, *A.latronum*, *A.peninsulae*, and *A.agrarius*) in the Asian Far East. Results from our analyses were different from this study in several ways. First, in their study, the cyt *b* haplotypes from Yunnan Province did not cluster with any other haplotypes from Sichuan Province, and they formed a single lineage [35]. However, in our study, individuals from Yunnan Province (populations 2 and 3) clustered together with individuals from Daocheng (population 18), Sichuan Province (Fig. 2). Second, haplotypes from Maerkang, Emei Mountain and Baoxing fell into one lineage [35], but phylogenetic tree in our study clearly showed that individuals from Maerkang (population 4) formed clade 2, and samples from Emei Mountian (population 1) and Jiajin Mountain (population 6) fell into subclade 1A and subclade 1C, respectively (Fig. 2). It is possible that their [35] limited numbers of individuals and sampling sites (individuals: 13 in Yunnan and 22 in Sichuan; sampling sites: 4 in Yunnan and 4 in Sichuan) caused these differences. Based on the topographic complexity and unique geologic history of the SEMTP, it is necessary to use more samples in this region to investigate the relationship between geologic events and local species’ evolutionary history.

*Apodemus draco* exhibits a high level of population structure in our study. Most individuals from the same or nearby sampling localities clustered together, with the exception of individuals from Gongga Mountain (in both clades 1 and 2) and individuals from Jiajin Mountain (in different subclades of clade 1). The results of multiple landscape analyses further support the geographic structuring of genetic diversity in this species. As shown in Figure 1, some big rivers and mountains would act like barriers, such as Yalong River, Dadu River, and Qionglaishan Mountain and Jiajin Moutain. But other rivers and mountains did not act as barriers, such as Daxiangling Mountain. Hongya County (population 9) and Emei Mountain (population 1) located at right side of Daxiangling Mountain, but their samples were clustered with samples from its left side (populations 6, 11, 12, 15, 16). Therefore, the phylogeographical structure of *A. draco* was very complicated due to the complex geologic history and topography of the SEMTP. However, an unexpected pattern was observed in clade 4. Individuals from the central part of Sichuan Province, Zhongguo (population 5), clustered together with samples from southern Sichuan Province, including Jinyang, Zhaojue, Yuexi, Jiulong and Shimian Counties (populations 7, 8, 10, 13, 14) and the Gongga Mountain (population 15), whereas the samples from Hongya County (population 9) and Emei Mountain (population 1), which were more close to the sampling sites in southern Sichuan, did not fall into clade 4 (Fig. 1). Further studies are needed to elucidate the unique pattern found in clade 4.

The estimated divergence times of *A. draco*’s main splits (2.20–0.55 Mya) were congruent with the periods of the Qingzang Movement (3.6–1.7 Mya) and Kun-Huang Movement (1.2–0.6 Mya) [9]–[11]. These two movements were known as the most intense uplift events in the Tibetan Plateau. These events shaped the geologic configuration of this area, making the topography of the plateau very complex [13]. Because small rodents are sensitive to the changing environment, possess limited migration abilities [22], [24], therefore, many phylogenetic and phylogeographic studies [3], [4], [14], [18], [22] have demonstrated that uplift events and environmental complexity played an important role in the diversification of local species. It is reasonable to suggest that the initial divergence of *A. draco* would have been shaped by the uplift events of the Tibetan Plateau, and high levels of genetic diversity have beeen maintained as a result of these events. Although the divergence times in our study was rough estimates, this conclusion was supported by Sakka *et al.* [35]’s study. In their work, the divergence time between the main lineages of *A.draco* corresponded to periods from 1.5–1.7 Mya to 2.0-2.2 Mya, and they further suggested that the complex land conditions could explain the divergence of *A. draco* in Sichuan Province.

The Tibetan Plateau underwent four or five glaciations during the Quaternary period [66], and the frequency and range in this plateau was less than that in other regions in Asia [66], [67]. According to geologic data, the extensive glacial period (EGP) on the Tibetan Plateau occurred during the middle Pleistocene (about 0.5 Mya), and continued until 0.17 Mya [66], [68], [69]. The last glacial period (LGP) occurred 0.08–0.01 Mya, in which the last glacial maximum (LGM) was 0.021–0.017 Mya [9], [70]. During the EGP, ice coverage could be permanent at high elevations and central regions of the Tibetan Plateau [69], [70], [71]. Conversely, the frequency of glaciers in the east was less than that in the west, and indeed, no large ice sheets were found in the SEMTP, so many continuously ice-free areas were likely to have existed in the SEMTP because of its lower elevation and the complicated geologic configuration [66], [67], [70], [71]. Consequently, many areas located in the SEMTP could provide suitable refugia, allowing *A. draco* to maintain stable population sizes.

Indeed, some recent phylogeographic studies have indicated that the large mountain ranges on the east edge of the Tibetan Plateau could provide substantial refugia for the local species during the Quaternary [19], [22], [72]. However, this did not mean the major glaciations in the Pleistocene did not have any effect on the evolution of *A. draco*. The estimated divergence time showed that the split between clade 1 and clade 2 occurred at 0.26 Mya, which fell into the EGP (0.5–0.17 Mya). Thus, it was possible that the largest glaciation further isolated these groups, which could promote the differentiation. Similarly, Zhan *et al.* [72]’s study showed that the major glaciations during the Pleistocene have had a major impact upon the evolution of the blood pheasant (*Ithaginis cruentus*).

The IBD and SAA results showed that there was no clear relationship between geographic distance and genetic distance in the central region (clade 1), although there were many huge mountains. It seems that there is a certain connection between the pattern in clade 1 and the regional historical demography. Indeed, two of the subclades in clade 1 experienced population expansions.

Subclade 1A was estimated to have experienced population expansions at about 0.052–0.013 Mya, after the EGP (0.5–0.17 Mya), and probably before the LGM (0.021–0.017 Mya). Most individuals of this subclade were collected from Hongya County (population 9). Its elevation is low, and the sampling site in this county (about 1800 m) was lower than other sampling sites (2450–3700 m) in subclade 1A. Thus, Hongya could have provided suitable habitats for species during the glacial period. During the population expansions, many individuals from nearby areas might have migrated into Hongya County. At the same time, some individuals in these regions could have dispersed into nearby areas. This expansion scenario could explain why several individuals from the Jiajin Mountain (population 6) appeared in subclade 1A.

The expansion of the subclade 1B was inferred to have happened at about 0.014-0.004 Mya, right after the LGM (0.021-0.017 Mya). Most individuals of subclade 1B were collected from Gongga Mountain (population 15). Gongga Mountain located at the eastern margin of the Tibetan Plateau, was an important glaciation centre in the eastern Tibetan Plateau. Here, remains of Quaternary glaciers are still widespread. Glacial accumulation and erosion landforms coexisted, which indicated this region has been glaciated many times [20]. The range and intensity of the Quaternary glaciers in this region were very different in different periods. All contemporary glaciers are located at high elevations (above 3000 m). During the early Pleistocene, no glacier developed in the GMR because the relative low elevation at that time. Glaciers developed during the mid-Pleistocene, but most of them occurred at valley heads or high altitudes [20]. The GMR experienced the most extensive glaciation at about 0.277 Mya [20]. As a result, many small areas in this region were suitable for species to inhabit during different periods. Additionally, many ancient and endemic species inhabited in this region [21], [23] (e.g. Flora: *Taxus chinensis*, *Kingdonia uniflora*, *Cordyceps sinensis*, *Cercidiphyllum japonicum*; Fauna: *Ailuropoda melanoleuca*, *Panthera uncia*, *Pantholops hodysoni*, *Rhinopithecus roxellanae*), leading to the conclusion that the GMR acted as a refuge during the cold periods [21], [22].

Another character of Gongga Mountain is its elevation is very large, ranging from less than 1000 m to more than 7000 m, so many types of micro-environments are available [20], [21], [73]. *Apodemus draco* has been found at elevations from 800 m to 4000 m, and it occupies different microhabitats, such as broad-leaved forests, mixed broadleaf-conifer forests, scrublands, and meadows [23], [74]. Therefore, *A. draco* could have found available microhabitats in high elevations or simply retreated to lower elevations. Consequently, based on the characteristics of *A. draco* and the geologic history of the GMR, *A. draco* could have survived in many parts of the GMR even during the EGP and LGM. This conclusion could explain why individuals from GMR fell into different evolutionary clades (Fig. 2).

Two recent studies found the similar pattern in the blood pheasant (*I. cruentus*) [73], Sichuan field mouse (*A. latronum*) and Chinese scrub vole (*Neodon irene*) [22]. But the Sichuan field mouse and Chinese scrub vole had to find suitable places at high elevations in the SEMTP instead of shifting elevation, because they are only found in high elevations [22], [23], [74]. Therefore, since there were two refugia for *A. draco* in clade 1 (Hongya county and GMR) in the central region, the discontinuous gene flow among different sampling regions could form the current observed pattern at clade 1.

### Conclusion

The present study demonstrated that the genetic differentiation of the South China field mouse’s population in the SEMTP not only was influenced by the complex geologic history and unique topography of the SEMTP, but also was influenced by the major glaciations in the Pleistocene. As a consequence, we hypothesize that its evolutionary history included two stages. First, an initial divergence would have been shaped by the uplift events of the Tibetan Plateau, and high genetic diversity was maintained by environmental and topographic complexity. Then, major glaciations in the Pleistocene added further complexity to its demographic history and genetic structure. The observed genetic differentiation pattern in this work is consistent with that of other recent studies focused on the eastern Tibetan Plateau [8], [19], [22], [72]. Our work, together with the studies from Fan *et al.* [22] and Zhan *et al.* [72], indicate that past geologic and climatic events on the eastern Tibetan Plateau could have similar effects on the regional historical demography of many animals in the region.
